# Ion Acceleration and D-D Nuclear Fusion in Laser-Generated Plasma from Advanced Deuterated Polyethylene

**DOI:** 10.3390/molecules191017052

**Published:** 2014-10-23

**Authors:** Lorenzo Torrisi

**Affiliations:** Dipartimento di Fisica e SdT, Università di Messina, V.le S.F. d’Alcontres 31, 98166 S. Agata, Messina, Italy; E-Mail: Lorenzo.Torrisi@unime.it; Tel.: +39-090-676-5052; Fax: +39-090-395-004

**Keywords:** D-D fusion, laser-generated plasma, time-of-flight measurements

## Abstract

Deuterated polyethylene targets have been irradiated by means of a 10^16^ W/cm^2^ laser using 600 J pulse energy, 1315 nm wavelength, 300 ps pulse duration and 70 micron spot diameter. The plasma parameters were measured using *on-line* diagnostics based on ion collectors, SiC detectors and plastic scintillators, all employed in time-of-flight configuration. In addition, a Thomson parabola spectrometer, an X-ray streak camera, and calibrated neutron dosimeter bubble detectors were employed. Characteristic protons and neutrons at maximum energies of 3.0 MeV and 2.45 MeV, respectively, were detected, confirming that energy spectra of reaction products coming from deuterium-deuterium nuclear fusion occur. In thick advanced targets a fusion rate of the order of 2 × 10^8^ fusions per laser shot was calculated.

## 1. Introduction

A high intensity laser interacting with dense matter generates, in vacuum, a non-equilibrium plasma exhibiting high temperature and density and able to drive ion acceleration at energies above 1 MeV per charge state. Thermal and Coulomb interactions contributing to the final ion velocity evidence higher values in a direction normal to the target surface, according to the Coulomb-Boltzmann-shifted energy distribution model [1]. Of particular interest is the proton acceleration both in Backward Plasma Acceleration (BPA) regime, irradiating thick targets, and in Target Normal Sheath Acceleration (TNSA) regime through thin foils developing hot directive plasmas on the rear of the illuminated side as argued in [2,3]. In the first case high ion yields, currents, and low kinetic energies, below 1 MeV per charge state, are attainable from thick targets, while in the second case low ion yields, due to the reduced thickness of the target, and high ion kinetic energies, above 1 MeV per charge state, can be produced.

During the past few years at the Prague Asterix Laser System (PALS) laboratory, a laser pulse of 300 ps duration, 1315 nm wavelength, and 10^16^ W/cm^2^ intensity, was used to accelerate deuterons using both the BPA and TNSA approaches to irradiating deuterated targets [4]. Different diagnostic techniques were employed to measure the energy of accelerated deuterons and that of the products of characteristic energy coming from the D-D nuclear fusion, according to the two equal probable reaction channels [5]:

D + D → T (1.01 MeV) + *p* (3.02 MeV) (1)

D + D → ^3^He (0.82 MeV) + n (2.45 MeV) (2)

The number of nuclear fusion events is high and it depends on the setting of laser parameters, irradiation conditions and target composition and geometry. These results are due mainly to the kinetic energy of the accelerated deuterons, near the value of the maximum D-D cross-section, more than to the plasma temperature [6]. The deuterated targets, irradiated by the short laser pulse, emit fast electrons, becoming positively charged and undergoing Coulomb explosion processes. As a consequence, ions are emitted and accelerated by high directive electric fields, and driven by the electron emission cloud mainly along the direction normal to the target surface [7].

**Figure 1:**
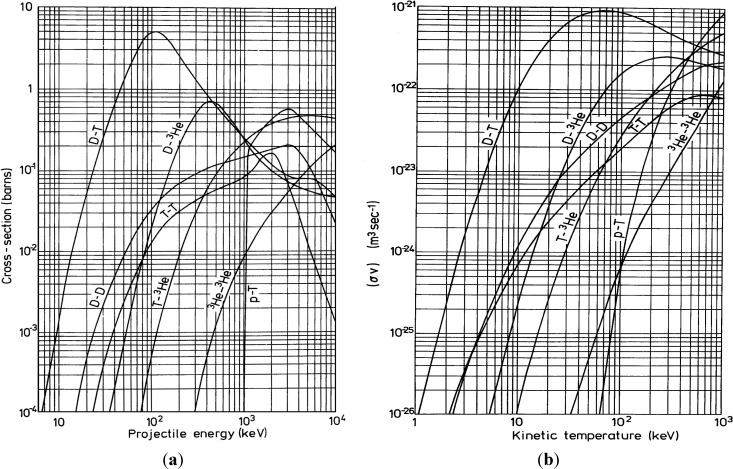
(**a**) D-D nuclear fusion cross-section *vs.* the deuterium energy and comparison with the cross sections for the D-T, D-^3^He, T-T, T-^3^He, ^3^He-^3^He and p-T fusion processes; (**b**) D-D fusion reactivity, in terms of the average of the fusion cross-section σ over the relative velocities *v*, *vs.* plasma temperature and in comparison with the fusion reactivity for the D-T, D-^3^He, T-T, T-^3^He, ^3^He-^3^He and p-T fusion processes, according to [8].

Figure 1a shows the plot of the D-D nuclear fusion cross-section σ *vs.* deuterium energy and comparison with the cross sections for the D-T, D-^3^He, T-T, T-^3^He, ^3^He-^3^He and p-T fusion processes. Figure 1b reports the D-D fusion reactivity, in terms of average of the fusion cross-section σ over the relative velocities *v*, *vs.* plasma temperature and in comparison with the fusion reactivity for the D-T, D-^3^He, T-T, T-^3^He, ^3^He-^3^He and p-T fusion processes, according to [8].

Figure 1a indicates that the maximum cross-section of the D-D fusion occurs at 3.0 MeV deuteron projectile energy, while Figure 1b shows that the D-D fusion reactivity, <σv> given in m^3^/s, increases with the plasma temperature up to saturation zones above 100 keV. The use of thin targets in TNSA regime and/or “advanced” thick targets in BPA regime produces high ion acceleration and hot plasma which enhances the number of fusion events per laser shot, as will be demonstrated by monitoring the characteristic protons and neutrons emitted from the D-D nuclear reaction and as will be discussed below.

## 2. Results and Discussion

**Figure 2:**
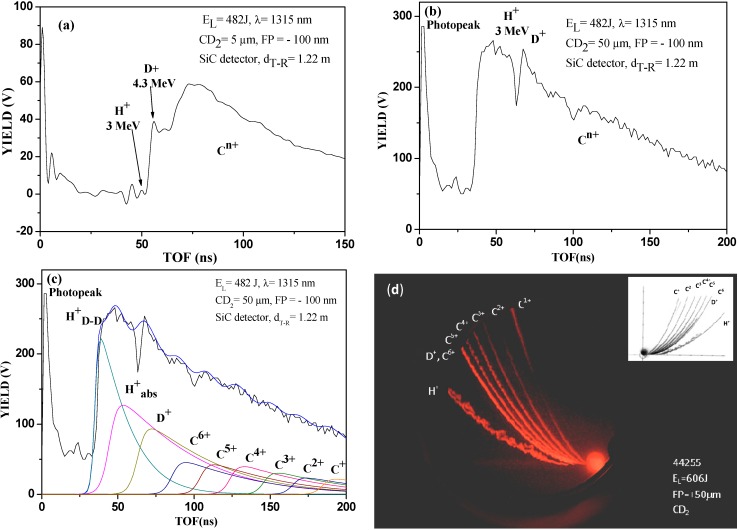
SiC TOF spectra relative to the laser irradiation of CD_2_ target thick 5 µm (**a**), and 50 µm (**b**), deconvolution of the ion yield with the different ion contributions (**c**) and Thomson Parabola spectrum obtained irradiating 5 µm deuterated polyethylene target in TNSA regime (**d**).

A typical result obtained during the first experiment, relative to the laser irradiation at 600 J of a thin CD_2_ foil in TNSA conditions, is reported in Figure 2 in terms of SiC TOF spectrum for a CD_2_ foil with a thickness of 5 µm (a) and 50 µm (b) and of charge to ion mass ratios detected through Thomson Parabola Spectrometry (d) [9]. The SiC spectra show a photopeak, used as starting signal, due to the photoelectric effect, followed by a plateau, due to electrons and their Bremsstrahlung detection, and different peaks owing to the detection of different ion species. The electrons are detected in the energy spectrum ranging from 1 keV to 100 keV. Higher energetic electrons may be there, but they are super-imposed on the photopeak and their energy tracking is not possible using SiC in TOF configuration. The faster ions are protons, followed by deuterium ions and by carbon ions, which velocity decreases with the increasing ion mass and decreasing ion charge state.

Irradiating under the same conditions CH_2_ (non-deuterated target) and CD_2_ (deuterated target), the two TOF spectra are different. In the first case protons are accelerated at an energy of 2.15 MeV, while in the second one deuterons, having a double mass, are accelerated to 4.3 MeV kinetic energy, in agreement with the electric field driving the ion acceleration. The CD_2_ target also contains absorbed hydrogen as contaminant and shows a small proton acceleration component. Moreover, due to the possible D-D fusion processes and to the (d, *p*) reaction channel, protons from such targets can be detected up to 3.0 MeV kinetic energy. Generally speaking, the yield of the contaminant species is lower than the yield of the compound elements, thus the ion peak assigned to accelerated deuterons coming from CD_2_ target is predominant with respect to that coming from the proton contribution.

The spectrum of Figure 2a indicates a high yield due to the deuteron acceleration and a lower peak, corresponding to protons at 3.0 MeV of kinetic energy, due to the contribution coming from the D-D nuclear fusion reaction in a 5 µm thin target. Carbon ions are accelerated at a mean energy of about 2.15 MeV per charge state, thus the higher ion charge state C^6+^ are acquired to a maximum energy of 12.90 MeV. The charge state distribution of carbon ions generally shows a decrease with the charge state due to their higher ionization potential, ranging between 11.3 eV to 490 eV for C^1+^ and C^6+^, respectively. Thus the maximum yield of the carbon peak is due to C^1+^ and C^2+^, however the SiC detector, exhibiting a response proportional to the ion energy released in the depletion region, produces a spectrum with a maximum signal that can be attributed mainly to the contribution of C^5+^ and C^4+^ ions.

The spectrum of Figure 2b indicates a higher yield due to protons of about 3.0 MeV kinetic energy, due to the contribution coming from the D-D nuclear fusion reaction in the 50 µm target, followed by deuterium plasma acceleration contribution. These results demonstrate that for thin films irradiated under TNSA conditions the emitted protons due to the (d, *p*) reaction channel are proportional to the film thickness. Figure 2c reports the deconvolution procedure performed using Coulomb-Boltzmann-shifted ion energy distributions for different detected ions [10].

Finally, the fourth spectrum of Figure 2d shows a typical TPS image indicating the parabola curves relative to protons (the lower one) and to six charge states of the carbon ions. The deuterium parabola is coincident with the C^6+^ parabola, due to the same charge to mass ratio; this parabola, in fact, has a higher light intensity with respect to the other C ion parabola contributions because the two parabolas are overlapped and cannot be separated. Even the proton parabola is thicker and larger than the others due to two proton components, coming from the plasma acceleration of hydrogen as contaminant absorbed in the deuterated target and having a maximum energy of 2.15 MeV and as protons coming from the D-D nuclear reaction production (protons generated at 3.0 MeV energy and emitted at energies lower than 3.0 MeV due to proton energy loss in the generated plasma). The inset of this spectrum reports a comparison of experimental parabola with the simulation ones obtained taking in consideration the real TPS geometry and the values of used magnetic and electric fields [11].

**Figure 3:**
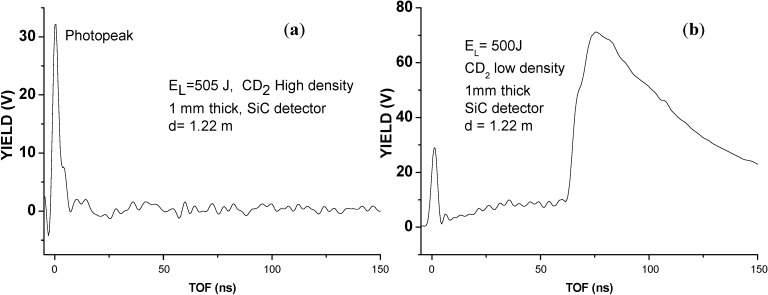
SiC TOF spectrum relative to laser irradiation of high density bulk deuterated polyethylene (**a**) and spectrum relative to the laser irradiation of 1 mm thick foam CD_2_ target, at low density (~9 mg/cm^3^), indicating a deuterium ion acceleration of about 3.15 MeV and protons detection at 3.0 MeV (**b**).

The second experimental run, obtained from laser irradiating of a CD_2_ target with an high density of 0.93 g/cm^3^ in BPA regime, has demonstrated that the D-D nuclear reaction doesn’t occur. The lack of occurrence of this reaction is due to the lower energy released for the backward directed deuterated ion accelerations that generally produces low kinetic energies of about 40 keV, in agreement with the measurement of proton acceleration from non-deuterated polyethylene giving 20 keV under the same experimental conditions, as confirmed by IC detectors. The SiC spectrum of Figure 3a, relative to this experiment, in fact, doesn’t show deuterium ions because, due to their low energy, these ions are stopped in the thin 200 nm in thickness metallic film of Ni_2_Si covering the semiconductor and used as metallic contact on the surface of the Schottky junction. The D-D fusion cross section is negligible at 40 keV of energy with respect to the value obtainable at 3.0 MeV, so that the nuclear fusion events are negligible in this case.

In the third experiment, conducted by using advanced targets, such as foam targets at low density (~9 mg/cm^3^) and high thickness (1–3 mm), a higher laser absorption occurs, deuteron acceleration is induced in both backward and forward directions and it was possible to detect deuterons at high kinetic energy, above 3.0 MeV, and protons, coming from D-D fusion processes at the characteristic 3.0 MeV energy. Figure 3b shows the SiC TOF spectrum relative to the laser irradiation of 1 mm thick foam CD_2_ target, at low density (~9 mg/cm^3^) indicating a deuterium ion acceleration of about 3.15 MeV and ion acceleration of 1.57 MeV per charge state, confirming the maximum carbon C^6+^ ion acceleration at 9.4 MeV. A signal due to protons at energy from 3.0 MeV up to values lower than 1 MeV is detected both using SiC and in TPS detectors.

This result demonstrated that advanced targets, such as the highly absorbent foam polyethylene, increase the laser energy absorbance generating higher temperature plasma with respect to that obtained irradiating high density polyethylene. The foam is converted into underdense hot plasma after the ionization wave traverses the foam. The initial part of the laser pulse is absorbed by the foam and produces an extended corona of low density plasma. Laser beam propagates through this plasma because its electron density is lower than the critical one losing both spatial and temporal coherence due to the forward scattering of the laser light on the plasma density perturbations induced by the laser itself.

**Figure 4:**
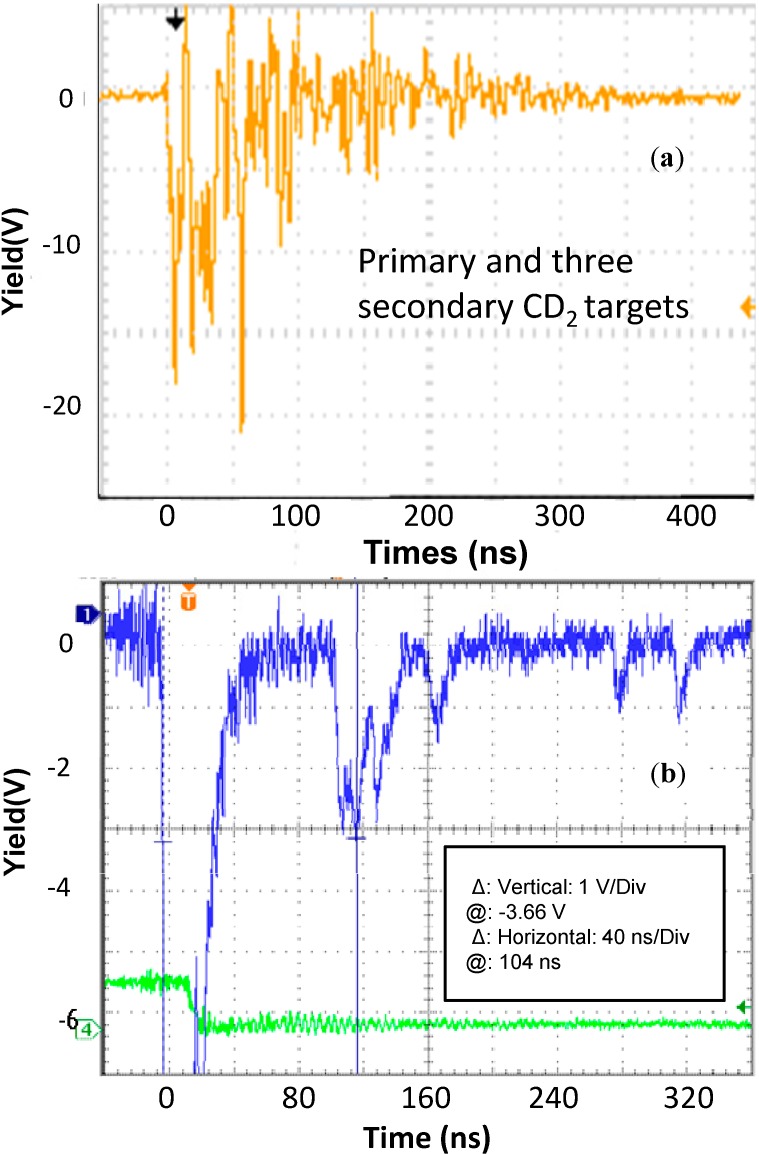
Plastic scintillator spectrum obtained for a detector placed in forward direction at a target distance of 1.25 m and for a laser irradiation of 5 µm CD_2_ high density target in TNSA regime (**a**) and scintillator spectrum for laser irradiation of the foam target, 3 mm in thickness, as primary target and by exposing to the backward accelerate deuteron flux three secondary CD_2_ thick targets (**b**).

In order to verify the D-D fusion occurrence, the plastic scintillators were employed to detect the neutrons emission. Two scintillators were placed in air, out of the vacuum chamber, and connected in time-of-flight mode with a fast storage oscilloscope. Figure 4a shows a typical spectrum obtained with a detector placed at 30° in the forward direction at a target distance of 1.25 m. In this case the target is CD_2_ with 5 µm in thickness, irradiated in TNSA regime, according to the first experiment. The spectrum is triggered with the laser shot and shows a first large negative peak, due to X-rays coming from the electron Bremsstrahlung, followed by a narrow peak located at 58 ns from the laser shot. It’s relevant to stress the consistency among this last one and neutron detection with a characteristic kinetic energy of 2.45 MeV coming from the D-D nuclear fusion processes.

Figure 4b shows a typical spectrum extracted in the case of the fourth experiment conducted using the foam target, 3 mm in thickness, as primary target and by exposing to the backward accelerated deuteron flux three secondary CD_2_ thick targets having 0.93 g/cm^3^ density and 1 mm thickness each. The secondary targets were placed at a low angle with respect to the normal to the target surface (~30°), in backward direction, at distances of 19 cm, 31 cm and 50 cm, respectively.

**Figure 5:**
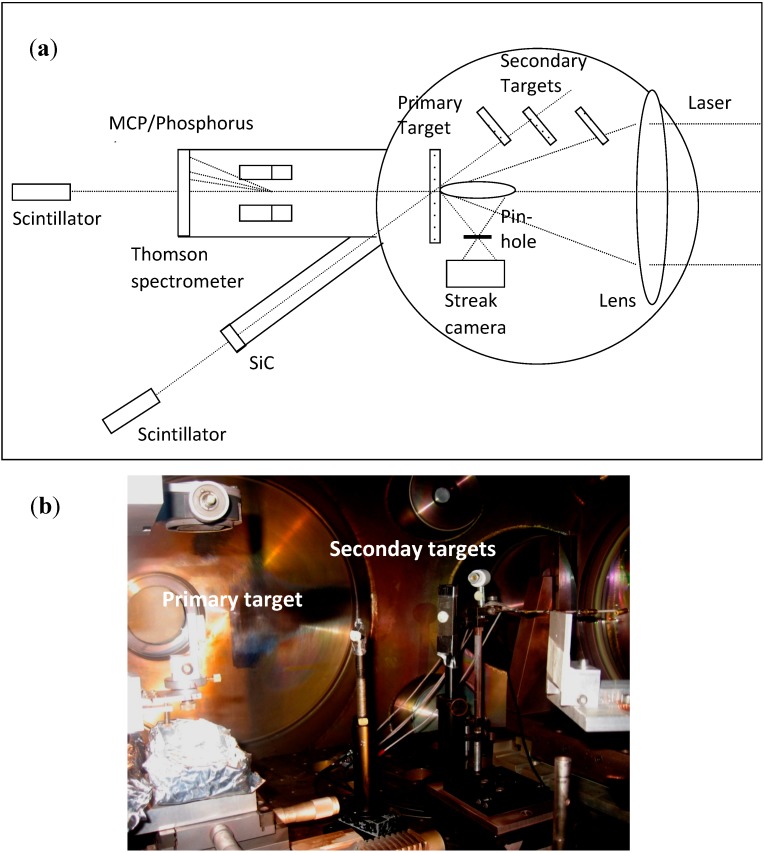
Sketch (**a**) and a photo (**b**) of the experimental set-up employed to perform the forth experiment by using the primary and the three secondary targets.

In this case the scintillator spectrum shows more details of the produced plasma emission radiations. In fact, not only the first peak owing to electron Bremsstrahlung is detected at the laser shot trigger, but also four peaks are detected at a TOF of 110, 123, 136 and 163 ns. The faster peak, due to the flight distance from the primary target to the scintillator of 2.4 m, indicates a neutron kinetic energy of 2.45 MeV. The other three peaks are compatible with characteristic neutrons coming from the three secondary targets. In fact, assuming the deuteron energy emitted towards the secondary targets to be 3.0 MeV, deuterons spend a time of 7.9, 12.9 and 21 ns to start from the primary target and to hit the three secondary targets, respectively. Thus correcting the TOF for this delay and using the correct flight distances from the secondary targets to the scintillator of 2.50, 2.68 and 3.0 m (see the scheme reported in Figure 5a), the measured neutron kinetic energy in the three cases corresponds to 2.45 MeV.

By analyzing the scintillator TOF spectra, the neutron peaks appear to have a TOF spread of about 8 ns around to the mean TOF value, corresponding to a neutron energy spread of about 300 keV. From the Wolle equation [12] these data permit us to determine, as a first approximation, the plasma temperature of fusing ions, according to the empirical relation:

kT (keV) = ∆E(keV)^2^/6806 (3)

from which it is possible to extrapolate an equivalent plasma temperature of about 13 keV.

This temperature is of the same order of magnitude but about a factor two higher with respect to the expected value determined by the plasma expansion velocity, calculable from the X-ray streak camera imaging, giving a plasma expansion of 300 µm in an exposition time of 1 ns, *i.e.*, an expansion velocity v_k_ = 3 × 10^5^ m/s.

**Figure 6:**
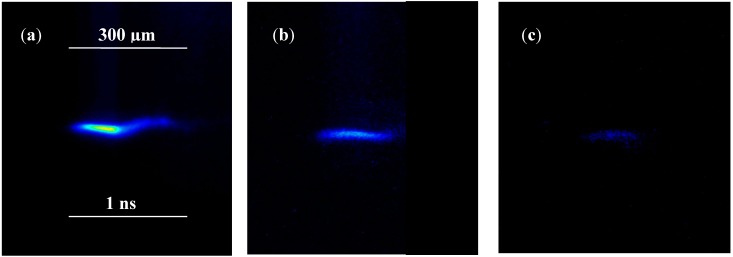
X-ray CCD streak camera images of CD_2_ plasma obtained irradiating in the same experimental conditions a high density deuterated polyethylene foil 50 µm in thickness in TNSA regime (**a**), a low density foam foil, 3 mm in thickness, (**b**) and a high density 3 mm deuterated foil irradiated in BPA regime (**c**).

Figure 6 reports three X-ray CCD streak camera images of the CD_2_ plasma achieved irradiating under the same experimental conditions a high density deuterated polyethylene foil 50 µm in thickness in TNSA regime (a), a low density foam foil, 3 mm in thickness (b) and a high density 3 mm deuterated foil irradiated in BPA regime (c). In the first case the plasma temperature is higher and the expansion velocity of 3 × 10^5^ m/s is calculable, while in the second case the expansion velocity is lower and in the third case the plasma temperature is very low.

The temperature corresponding to the maximum expansion velocity, as a first approximation, is evaluated as:

kT (eV) = mv_k_^2^/γ (4)

where m is the carbon mass and γ is the adiabatic coefficient (γ = 1.67 for monoatomic species). It corresponds to 6.4 keV for the measured expansion velocity in vacuum. At this temperature the fusion reactivity, in terms of average <σv>, has a low value of about 3 × 10^−25^ m^3^/s, as reported in the plot of Figure 1b.

As a first approximation it is possible to assume that the electron and ion temperatures are the same, but really such plasmas are in non-equilibrium conditions and the electron and ion temperatures can be different. In fact, in the brief laser pulse duration, due to the different electron and ion mobility and to the different collision frequency of the two species, electrons may be in about thermal equilibrium but ions not and these last may have lower temperatures with respect to electrons.

An evaluation of the number of the fusion events per laser shot was obtained using different approaches. The first was evaluated from the (d, *p*) reaction channel by measuring the 3.0 MeV proton flux using the SiC and the IC detectors.

For the CD_2_ target at the maximum thickness of 50 µm employing in TNSA regime, the number of protons was measured taking into account the proton current in the SiC detector and in the ion collector with respect to the solid angle subtended by the detector per laser shot, obtaining the yield of:

Y*_p_* = (V/R)(∆t/e)(Ω/∆ Ω) ~ 10^8^ protons/4π sr per laser shot (5)

being V the voltage measured on the fast storage oscilloscope (2 V), R the input resistance (1 M Ω), ∆t the duration of the proton bunch (10 ns), *e* the electron charge, and ∆ Ω/Ω the solid angle subtended by the detector normalized to the total one.

The second approach was evaluated from the (d, n) reaction channel by measuring the 2.45 MeV neutron flux using the bubble dosimeters which have released, on the average, 1 bubble for laser shot hitting 50 µm thickness CD_2_ target. Due to the dosimeter calibration of 4 bubble/µSv, this means that the dose received by a single dosimeter per laser shot is 0.25 µSv. Since the dosimeter sensitive volume has a mass M = 20 g and its solid detection angle is dΩ = 1 msr, taking in consideration the low detection efficiency ε ~ 10^−7^ of the dosimeter, this means that the number of neutrons with the characteristic energy E_n_ = 2.45 MeV is given by:

Y_n_ = (0.25 µSv) M/εE_n_ (d Ω/Ω) = 1.6 × 10^8^ neutrons/4π per laser shot (6)

A third approach was extracted from the (d, n) reaction channel by measuring the neutron flux using the CR39 track detectors exposed to the laser-generated plasma emission though a thin absorber of aluminum foil under a solid detection angle of 5 × 10^−4^ sr in forward direction. The measurements of these neutron fluxes have been published in a previous paper [13]. Using a similar experiment, they indicated a neutron flux ranging between 1.6 × 10^8^ and 5 × 10^8^ neutrons/sr. The above-mentioned value is comparable to other similar experimental measurements performed with laser-generated plasma at PALS [14].

## 3. Experimental Section

Experimental measurements were performed at the PALS laboratory in Prague using the 3-TW Asterix Iodine laser operating in single pulse at 1,315 nm wavelength, 300 ps pulse duration, 70 µm laser spot diameter and 10^16^ W/cm^2^ pulse intensity. The laser beam profile is Gaussian and no prepulse was used in this experiment.

Measurements were performed on irradiated normal incidence thick and thin films of (CD_2_)_n_ polyethylene, at high density (0.93 g/cm^3^) and foam at very low density (9 mg/cm^3^). The foams were prepared at the Physics Department of Messina University using Physical and Chemical processes starting from deuterated polyethylene powder. These foams have an open-cell-structured, known as reticulated foams, containing micrometric pores that are connected to each other and form an interconnected network that is relatively soft. The low density of these foams made them excellent optical light absorbers. The light hitting the material has very low reflection and transmission because it is trapped in the pores and it is scattered by the pore surfaces and absorbed in all directions inside the polymer. The foam polyethylene was obtained by peculiar chemical processes inducing chain cross-linking in gas phase and producing high porosity bulk material. (CD_2_)_n_ targets were irradiated as flat sheets with thicknesses from 5 µm up to 3 mm.

Plasma was monitored both in backward direction (from the laser irradiated front face) and in forward direction (from the rear face of the irradiate target).

Ion Collectors (IC), SiC detectors and plastic scintillators (NE-102A) were employed to detect photons, electrons and ions. Such detectors were placed at different angles with respect to the normal target surface and at different distances from the target, in forward and backward directions, and used in Time-Of-Flight (TOF) configuration, by acquiring their signal with a fast storage oscilloscope.

The IC are represented by a Faraday cups with a biased suppressor grid; the SiC uses a Schottky diode junction with 80 µm depletion layer covered by 200 nm Ni_2_Si metallic electrode; the plastic scintillators, based on polyvinyltoluene, exhibit very fast response times and are coupled to a fast high-gain photomultiplier tube capable of resolving the first photoelectrons of a light pulse produced by the scintillator medium. Scintillators cannot detect plasma electrons and ions because are placed in air, output from the vacuum chamber. In such conditions they are sensitive only to the gamma rays and to neutrons.

A Thomson Parabola Spectrometer (TPS), coupled with a Multi-Channel-Plate (MCP), a phosphorous screen and a CCD camera, was placed in forward direction along the normal to the target surface (0°). The laser focal position (FP) on the target surface was controlled though an X-ray CCD streak camera by observing the plasma with 2 ns exposition time through 50 µm pinhole with axes is orthogonal to the target normal direction. More details on the diagnostic methods employed are provided in the literature [9,11,15]. Micrometric step-motors allowed fine adjustment of the focal position in order to check the self-focusing conditions obtainable for distances of the order of 100 µm in front of the target surface [16].

Four main experiments were performed as described below:

(i) the first one by irradiating in TNSA regime a 5 µm thickness CD_2_ foil;
(ii) the second one was conducted by irradiating in BPA regime 1 mm thick CD_2_ target having 0.93 g/cm^3^ density;
(iii) the third one by irradiating a CD_2_ foam target having a density of about 9 mg/cm^3^ and a thickness of 1–3 mm;
(iv) the fourth one was conducted using the previous foam target in BPA regime as primary target and by exposing to the backward flux of accelerated deuterium ions to three secondary CD_2_ thick targets having 0.93 g/cm^3^ density and 1 mm thickness (secondary targets).

Characteristic protons and neutrons were detected using SiC devices placed at 60–122 cm distance from the target, in vacuum, and two plastic scintillators placed both in forward direction, one at 1.25 m from the target and at 30° angle with respect to the target normal direction and another placed at 2.4 m distance and at 0° (along the normal direction). Scintillators operated in current mode and use a lead shielding to reduce the background noise. Moreover, track detectors using CR39 and calibrated bubble neutron dosimeters, with a sensitivity of 4 bubbles/µSv (Bubble Technology Industries, Chalk River, ON, Canada [17]), were employed to detect characteristic protons and neutrons emitted from the nuclear fusion processes. Bubble detectors have zero gamma sensitivity, require no power, are reusable, and have an isotropic angular response. Figure 5 shows a scheme (a) and a photo (b) of the experimental set-up employed to perform the forth experiment by using the primary and the three secondary targets.

## 4. Conclusions

Based on the detected characteristics proton and neutron yields produced per laser shot by the nuclear processes and by assuming their emission to be isotropic, the number of evaluated fusion events is of the order of some units of 10^8^, in agreement with previous similar measurements reported in literature. However instabilities in laser pulse energy, small differences in the irradiation conditions, and target non-uniformities produce variations on the laser fusion processes that may be evaluated at round 30% or more with respect to the given mean value. In general measurements have reported that the number of fusion events is proportional to the laser pulse intensity, thus scaling approximately with the proportionality law to the Iλ^2^ parameter it can be increased with the laser intensity [7].

Some relevant issues of the presented investigations concern:

(1) The use of foam deuterated polyethylene target, 3 mm in thickness having very low density, favors the generation of nuclear fusion events with respect to the use of high density polyethylene. This result may be attributed to the higher laser absorbance at the used laser wavelength. The transmission in PE at 1315 nm wavelength, in fact, is about 90% [18], thus low laser energy is deposited in the thin target and transferred to the plasma. The absorbance in low density foam polyethylene, instead, increases of about a two factor or more [19], thus it permits to enhance the laser energy transfer to the generated plasma, *i.e.*, increasing both the plasma temperature and the deuteron ion acceleration, enhancing the probability to induce D-D fusion processes.
(2) The focal position of the laser with respect to the target surface is crucial. Measurements demonstrated that the maximum fusion events are obtained for a focal position between 0 and −100 μm, decreasing significantly out of this range. This result indicates that the high number of fusion event is obtained for high laser intensities at which the focal spot on the target surface is 70 μm diameter, as occurs for FP = 0 μm, or lower. Conventionally, negative FP means that the laser is focused before the target surface; in such conditions the preplasma increases the refraction index of the medium where laser propagates producing a further focalization of the laser on the target surface (self-focusing effect as argued in Ref [16]) reducing the spot size to dimensions comparable with the laser wavelength and generating non-linear phenomena.
(3) The plasma diagnostics employed in this experiment allow one to deduce the plasma temperature which was evaluated to be about 6 keV. At this temperature the D-D fusion reactivity is <σv> = 3 × 10^−25^ m^3^/s, thus, assuming the deuteron ions to be accelerated to 4 MeV, by valuing their mean velocity the calculable mean cross section is <σ> = 1.53 × 10^−4^ barns, a value three orders of magnitude lower with respect to that of 0.2 barns relative to the D-D projectile cross-section. Thus, this result indicates that the D-D fusion events are mainly due to the laser driven ion acceleration in plasma than by thermal processes driven by the Boltzmann velocity distributions in hot plasma.
(4) The estimated fusion events of about 2 × 10^8^ per laser shot are indicating the number of deuterium nucleus participant to the fusion processes. Taking into account that in the case of a 50 μm thick target at 0.93 g/cm^3^ density, irradiated with 70 μm spot diameter, the number of vaporized deuterium ions is of the order of 2.3 × 10^17^, this means that only a very low fraction of the deuterium atoms in the target participate in the fusion events. This is evidence of the need for further investigations to enhance the number of events obtainable from such experiments.
